# Dr. Dyce, on a Case of Polydipsia

**Published:** 1800-06

**Authors:** William Dyce

**Affiliations:** Aberdeen


### To Dr. BRADLEY.

Dear Sir,

Cases of Polydipsia happening so rarely, it is not to be wondered at if practitioners in general are deficient in their knowledge of the manner of treating such complaints, when they actually occur in practice. I, for one, confess myself ignorant of the method in which such a case ought to be treated; for having never met with it as an original disease, I was almost induced to believe that it could only happen in consequence of some other existing complaint, as fevers, &c. On these occasions, thirst is a very common attendant, and is easily removed by curing the primary disease; but when it happens without any other complaint or symptom to direct us to the cause, it requires some knowledge of similar cases to enable one to prescribe effectually.—The following being a case in point, I beg leave to request your opinion on the subject, presuming that, from your extensive practice, you may have met with something of the like kind, and therefore may be able to direct me in the proper mode of treatment, which shall be implicitly followed. *

A lady, some months ago, applied to me for advice, on account of a very extraordinary thirst which she at that time laboured under, attended with no other symptom of disease, excepting that she was now and then affected with what she termed a weakness of her nerves. Her appetite for food has never been in the smallest degree impaired; on the contrary, since she has been affected in this way, it has been rather better than before: her bowels, at the commencement, were not perfectly regular, but, by means of some gently aperient medicines, this was soon removed; her tongue, during the whole period, has been quite clean; and the only medicines she has taken were of the aperient and tonic kind; an emetic might have been proper, but the bad effects produced by one taken some years ago, deterred me from proposing it at this time.

Having asked her a number of questions, relative to her former habit of body, &c. I learned that the catamenia stopped about seven years ago; and about, or rather before, that

* The Editors are convinced that this Case, communicated to Dr. Bradley as a friend, ought to be laid before the Public; and that the answers of their learned Correspondents will be far more satisfactory to Dr. Dyce and their other Readers, than that of any individual.

time, ſhe was ſeized with an uncommon ſpitting, which laſted nearly eight months, after which time ſhe was quite well. The ſmell of what was ſpit up was extremely fœtid; and in the courſe of a day, two or three handkerchiefs would have been uſed in conſequence. About two years ago the ſpitting again returned, but continued for a ſhort time, as it was only in a trifling degree: ſince that time ſhe has kept in a tolerably good ſtate of health till Auguſt laſt, at which time, being in the country, ſhe eat about fifty or ſixty cherries in a forenoon, and complained of being rather uneaſy, for which ſhe ſwallowed about half a glaſs of brandy, and was relieved. She eat her dinner, &c. juſt as uſual, but next day the thirſt commenced, and has continued much in the ſame way ever ſince. The quantity neceſſary for her drink, in twenty-four hours, may amount to about three quarts, or at moſt one gallon; at the ſame time, ſhe ſays, that an equal quantity of urine is ſecreted. About a month after ſhe was affected in this way, ſhe was ſeized with a very violent tooth-ach (her teeth being moſtly carious); ſhe applied a bliſter behind the ear, which, together with ſome doſes of laudanum, to the amount of twenty-five, ſeldom more than thirty drops, to produce reſt, procured her relief; during this period, which was about ten days, ſhe had little or no thirſt, but whenever the pain ceaſed the thirſt returned. For ſome days paſt ſhe has been taking a few drops of laudanum every night at bed time (not more than twelve), from which ſhe thinks that ſhe has found ſome relief, not in her thirſt, but in being leſs *nervous*. She has alſo complained, within theſe few days, of a ſlight degree of cramp in the muſcles of the left leg and foot, attended with ſome heat in the palms of her hands; but theſe being in no great degree, have not been much attended to. Theſe, I think, are all the particulars that I have been able to collect, excepting that, in her outward appearance, ſhe is rather thinner; and if, from the ſtatement which I have now laid before you, you can adviſe in courſe what is proper to be done in this caſe, it will confer an additional obligation on,

Dear Sir,

Your already obliged,

And very obedient humble ſervant,

*Aberdeen, May* 3, 1800.

WILLIAM DYCE.

